# Autocrine and paracrine motility factors and their involvement in invasiveness in a human oral carcinoma cell line

**DOI:** 10.1038/sj.bjc.6690587

**Published:** 1999-08

**Authors:** R Hasina, K Matsumoto, N Matsumoto-Taniura, I Kato, M Sakuda, T Nakamura

**Affiliations:** Division of Biochemistry, Biomedical Research Center, Osaka University Medical School,Suita, 565-0871, Japan; Second Department of Oral and Maxillofacial Surgery, Osaka University Faculty of Dentistry, Osaka, Suita, 565-0871, Japan

**Keywords:** cell motility factor, HGF, TGF-α, TGF-β, tumour invasion, tumour–stromal interaction

## Abstract

Invasive potentials of malignant cancer cells are regulated by cell motility factors. To examine the regulation of motility and invasiveness in oral squamous carcinoma, we investigated autocrine- and/or paracrine-acting cell motility factors, using a newly established human cell line (IF cells) from oral squamous cell carcinoma, which has highly invasive and metastatic characteristics. Conditioned medium derived from IF cells stimulated cell scattering and migration of GB-d1 gallbladder carcinoma cells, indicating that IF cells secreted cell motility factors. Using antibodies, IF-derived cell motility factors proved to be transforming growth factor (TGF)-α and TGF-β1. Antibodies against TGF-α and TGF-β1 inhibited autonomous migration of the IF cells. On the other hand, in vitro invasion of IF cells was strongly enhanced by hepatocyte growth factor (HGF) but only slightly by TGF-α and TGF-β1. The conditioned medium from fibroblasts enhanced in vitro invasion of IF cells, an event abrogated by anti-HGF antibody, but not by antibodies against TGF-α and TGF-β1. Importantly, IF cells secreted a factor inducing HGF production in fibroblasts and the factor was identified as interleukin-1, which means that a mutual interaction exists between tumour cells and fibroblasts, as mediated by the HGF/HGF-inducer loop. These results indicate that IF cells utilize TGF-α and TGF-β1 as autocrine-acting motility factors and HGF as a paracrine-acting motility factor, and that invasiveness of IF cells is particularly stimulated by HGF derived from stromal fibroblasts. Utilization of multiple cell motility/invasion factors that act in distinct pathways may confer highly invasive and metastatic potentials in IF oral squamous carcinoma cells. © 1999 Cancer Research Campaign


*British Journal of Cancer* (1999) **80**(11), 1708–1717
© 1999 Cancer Research Campaign
Article no. bjoc.1999.0587

### Autocrine and paracrine motility factors and their involvement in invasiveness in a human oral carcinoma cell line

**R Hasina[1,2], K Matsumoto[1], N Matsumoto-Taniura[1], I Kato[2], M Sakuda[2] and T Nakamura[1]**

[1]Division of Biochemistry, Biomedical Research Center, Osaka University Medical School, Suita, Osaka 565-0871, Japan; [2]Second Department of Oral and Maxillofacial Surgery, Osaka University Faculty of Dentistry, Suita, Osaka 565-0871, Japan

**Summary** Invasive potentials of malignant cancer cells are regulated by cell motility factors. To examine the regulation of motility and invasiveness in oral squamous carcinoma, we investigated autocrine- and/or paracrine-acting cell motility factors, using a newly established human cell line (IF cells) from oral squamous cell carcinoma, which has highly invasive and metastatic characteristics. Conditioned medium derived from IF cells stimulated cell scattering and migration of GB-d1 gallbladder carcinoma cells, indicating that IF cells secreted cell motility factors. Using antibodies, IF-derived cell motility factors proved to be transforming growth factor (TGF)-α and TGF-β1. Antibodies against TGF-α and TGF-β1 inhibited autonomous migration of the IF cells. On the other hand, in vitro invasion of IF cells was strongly enhanced by hepatocyte growth factor (HGF) but only slightly by TGF-α and TGF-β1. The conditioned medium from fibroblasts enhanced in vitro invasion of IF cells, an event abrogated by anti-HGF antibody, but not by antibodies against TGF-α and TGF-β1. Importantly, IF cells secreted a factor inducing HGF production in fibroblasts and the factor was identified as interleukin-1, which means that a mutual interaction exists between tumour cells and fibroblasts, as mediated by the HGF/HGF-inducer loop. These results indicate that IF cells utilize TGF-α and TGF-β1 as autocrine-acting motility factors and HGF as a paracrine-acting motility factor, and that invasiveness of IF cells is particularly stimulated by HGF derived from stromal fibroblasts. Utilization of multiple cell motility/invasion factors that act in distinct pathways may confer highly invasive and metastatic potentials in IF oral squamous carcinoma cells.

**Keywords:** cell motility factor; HGF; TGF-α; TGF-β; tumour invasion; tumour–stromal interaction

The transition from in situ tumour growth to invasive and metastatic disease is initially characterized by the potential of the tumour cells at the primary site to cross tissue barriers and to invade local tissues. In case of malignant tumours originating from epithelial tissue, cells in the primary tumour lose adherent cell–cell interaction, create a pathway through basement membrane and surrounding stroma, and migrate through the pathway it has created. Thus, degradation of local extracellular matrix components and accompanying cellular movement are particular characteristics associated with invasive tumours.

Studies done in the past decade revealed a unique group of cell motility factors and that many growth factors and ligands for receptor tyrosine kinases share motogenic activity (enhancement of cell motility) (reviewed in Stoker and Gherardi, 1991; Wright et al, 1993; Levine et al, 1995; Chicoine and Silbergeld, 1997). Scatter factor was originally identified as fibroblast-derived motility factor for epithelial cells (Stoker and Perryman, 1985), while subsequent purification and characterization revealed this factor to be identical to hepatocyte growth factor (HGF) (Gherardi et al, 1989; Weidner et al, 1990, 1991; Furlong et al, 1991; Konishi et al, 1991; Naldini et al, 1991). HGF was originally identified and cloned as a mitogenic polypeptide for hepatocytes (Nakamura et al, 1984, 1989; Russell et al, 1984; Miyazawa et al, 1989). Several growth factors, initially identified as mitogenic polypeptides, also affect motility of a wide variety of cells, including platelet-derived growth factor (PDGF), insulin-like growth factor-I (IGF-I), IGF-II, basic fibroblast growth factor (bFGF), transforming growth factor (TGF)-α and TGF-β1 (also see above reviews). These cell motility factors play important roles in regulating cell movement in physiological and pathological processes, including embryogenesis, tissue repair, inflammation as well as tumour invasion (reviewed in Levine et al, 1995; Chicoine and Silbergeld, 1997; Wehrle-Haller and Weston, 1997; Birchmeier and Gherardi, 1998).

Migration and invasion of cancer cells are particularly regulated by cell motility factors which act in autocrine- and/or paracrine-related manner. Establishment of an autocrine loop of growth factors is involved in tumorigenic transformation and progression to malignant cancer cells (Sporn and Roberts, 1985; Ullrich and Schlessinger, 1990; Wright et al, 1993; Levine et al, 1995; Silletti and Raz, 1996; Chicoine and Silbergeld, 1997). In addition to these factors, participation of paracrine factors have been implicated in tumour invasion and malignant progression, as based on the notion that growth and invasive potentials of carcinoma cells are influenced through interactions with host stromal cells (van den Hooff, 1988; Matsumoto et al, 1989; Camps et al, 1990; Wernert, 1997). HGF is a mesenchymal- (or stromal-)derived paracrine factor which affects cell growth, cell motility and morphogenesis of a wide variety of cells, including malignant ones (Jiang et al, 1993; Matsumoto and Nakamura, 1997; Birchmeier and Gherardi, 1998). In vitro, HGF stimulates migration or

*Received 19 October 1998*
*Revised 16 February 1999*
*Accepted 17 February 1999*

*Correspondence to:* T Nakamura invasion of various types of carcinoma cells (Weidner et al, 1990; Matsumoto et al, 1994; Shibamoto et al, 1994; Jeffers et al, 1996; Bennett et al, 1997; Inoue et al, 1997; Nakamura et al, 1997; also reviewed in Jiang et al, 1998) and autonomous activation of cMet/HGF receptor through the autocrine loop of HGF confers invasive and metastatic potential of tumour cells (Jeffers et al, 1996).

Oral squamous cell carcinomas are generally malignant tumours, with invasive and metastatic potentials. We established a distinct tumour cell line (IF cells) from a patient with oral squamous cell carcinoma. These IF cells are highly invasive and metastatic when implanted into athymic mice and thus IF cells retain invasive and metastatic potential (manuscript in preparation). We hypothesized that cell motility and invasiveness of IF cells might be regulated by cell motility factors which act in an autocrine or paracrine manner. In the present study, we identified paracrine and autocrine cell motility factors in this tumour cell line. Therefore, IF oral squamous cell carcinoma cells utilize both autocrine- and paracrine-acting cell motility factors and utilization of these multiple factors presumably confers invasive and metastatic potential in this malignant oral squamous carcinoma cell line.

### MATERIALS AND METHODS

### Materials

Mouse monoclonal antibodies against human TGF-α and TGF-β1 were obtained from Calbiochem, Calbiochem-Novabiochem Int. (Cambridge, MA, USA) and TAGO Products (Burlingame, CA, USA) respectively. Polyclonal antibody against HGF was prepared from the serum of a rabbit immunized with human recombinant HGF and 1 μg $ml^{-1}$ anti-human HGF IgG completely neutralized the biological activities of 1 ng $ml^{-1}$ of human HGF (Matsumoto et al, 1996). Human recombinant TGF-α was kindly provided by Genentec (San Francisco, CA, USA) and TGF-β1 was purified from human platelets (Okada et al, 1989). HGF was purified from the culture media of Chinese hamster ovary cells transfected with an expression plasmid containing human HGF cDNA (Nakamura et al, 1989). Human recombinant interleukin-1 receptor antagonist (IL-1 RA) was obtained from R & D Systems Inc. (Minneapolis, MN, USA). Antibody against human PDGF was obtained from Promega (Madison, WI, USA).

### Cell culture

IF cells were originally established from a patient with squamous cell carcinoma of gingiva of the lower jaw. The cells were cultured in α-modified Eagle's medium (α-MEM) supplemented with 10% fetal bovine serum (FBS). MDCK clone 3B (Madin-Darby Canine Kidney) epithelial cells and GB-d1 human gallbladder carcinoma cells were kind gifts from Dr R Montesano (University of Geneva) and Dr H Shimura (Fukuoka University) respectively. MDCK and GB-d1 cells were cultured in Dulbecco's modified Eagle's medium (DMEM) supplemented with 10% FBS. Human dermal and oral fibroblasts were initially allowed to proliferate outward from tissue obtained during surgery and the cells were cultured in DMEM supplemented with 10% FBS.

### Assay for cell growth and scattering

IF cells were plated at 500 cells $cm^{-2}$ in 12-well plates in α-MEM supplemented with 10% FBS and cultured for 24 h. The culture media was replaced by α-MEM containing 1% FBS and the cells were cultured in the absence or presence of 10 ng $ml^{-1}$ HGF, TGF-α or TGF-β1 for 72 h. The number of cells was counted using a Coulter counter after dissociation by trypsin.

For cell scattering assay, MDCK or GB-d1 cells were plated at $2.5 \times 10^3$ cells $cm^{-2}$ in DMEM containing 10% FBS and cultured for 24 h. The conditioned medium from IF cells or growth factors were added, cells were further cultured for 24 h, placed under a microscope and photographed.

### Measurement of HGF production

Human skin or oral fibroblasts were seeded on 48-well plates at a density of $5 \times 10^4$ cells $cm^{-2}$ and cultured for 24 h. After replacing the media with fresh DMEM supplemented with 1% FBS and 2 μg $ml^{-1}$ heparin, appropriate amounts of cytokines or the conditioned medium from IF cells was added and the cells were cultured for 24 h. The concentration of HGF in culture media was measured by enzyme-linked immunoabsorbent assay (ELISA), using a kit (Institute of Immunology Co. Ltd, Tokyo, Japan).

### In vitro migration assay

Migration of tumour cells was carried out using Transwell chamber equipped with a filter membrane with 8-μm pores (Corning Costar Co., Cambridge, MA, USA). Tumour cells were plated at $5 \times 10^4$ cells $cm^{-2}$ in the upper compartment of the chamber, and cultured for 24 h, fixed in 70% ethanol and stained with haematoxylin and eosin. The number of cells migrating to the undersurface of the membrane through the pores was counted, as viewed microscopically. Six microscopic fields were randomly selected for cell counting. To examine the inhibitory effects of specific antibodies on migration of tumour cells, antibodies against TGF-α, TGF-β and HGF were added at 10 μg $ml^{-1}$.

### In vitro invasion assay

In vitro invasion of tumour cells was measured using the Biocoat Matrigel Invasion Chamber (Becton Dickinson Labware, Bedford, MA, USA). In this apparatus, Matrigel basement membrane components are reconstituted on a filter membrane with 8-μm pores. The cells were plated at a density of $10^5$ cells $cm^{-2}$ on the Matrigel in the upper compartment of the chamber and cultured for 48 h. The number of cells that invaded the undersurface of the membrane was counted, as described above. To examine the extent of invasion of IF cells into collagen gel matrix, the tumour cells were embedded in collagen gel (Type I Collagen, Nitta gelatin, Osaka, Japan) at $5 \times 10^3$ cells $ml^{-1}$, and cultured for 7 days. The culture medium was changed every second day.

### RESULTS

### Production and identification of motility factors from IF cells

IF cells were established from a lymph node metastasis of poorly differentiated squamous cell carcinoma which had primarily developed in the lower jaw. Histologically the tumour was highly infiltrative and invaded the capsular sheath of metastasized lymph nodes. When IF cell were subcutaneously implanted in the

© 1999 Cancer Research Campaign

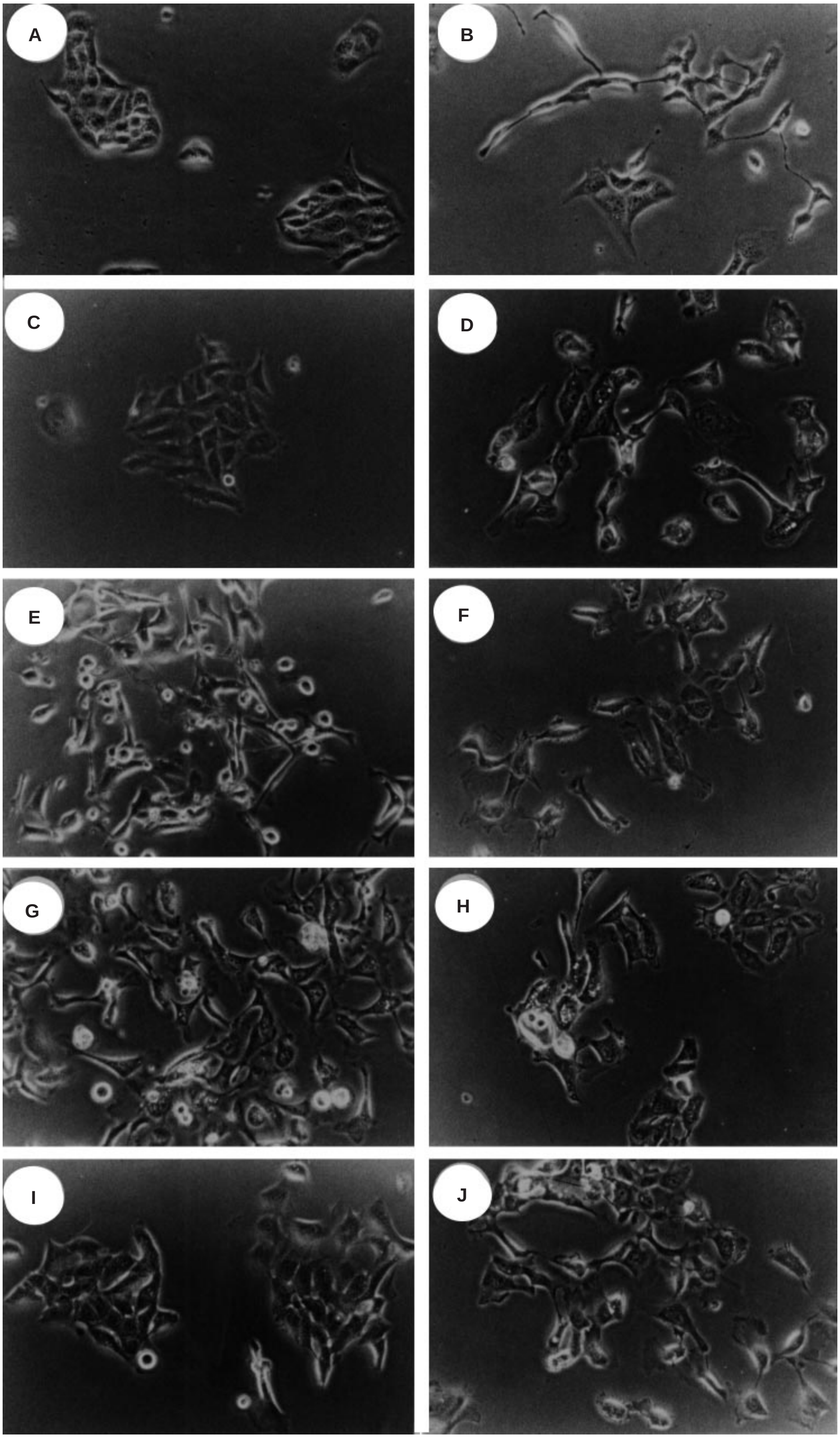

**Figure 1** Cell scattering induced by conditioned medium from IF cells and growth factors. Cell scattering was examined using MDCK renal epithelial cells (**A, B**) and GB-d1 gallbladder carcinoma cells (**C–J**). These cells were cultured in the absence (**A, C**) or presence of IF-derived conditioned medium (IF-CM) (**B, D**), TGF-α (**E**), TGF-β1 (**F**), HGF (**G**), IF-CM + anti-TGF-α IgG (**H**), IF-CM + anti-TGF-β IgG (**I**), or IF-CM + anti-HGF IgG (**J**). IF-CM was added at 50% (v/v) to these cultures, growth factors were at 10 ng $ml^{-1}$ and IgGs were at 10 μg $ml^{-1}$. These antibodies respectively neutralized cell scattering activity of 10 ng $ml^{-1}$ TGF-α, TGF-β1 and HGF and normal IgG had no effect on cell scattering activity of IF-CM (not shown)

 *© 1999 Cancer Research Campaign*

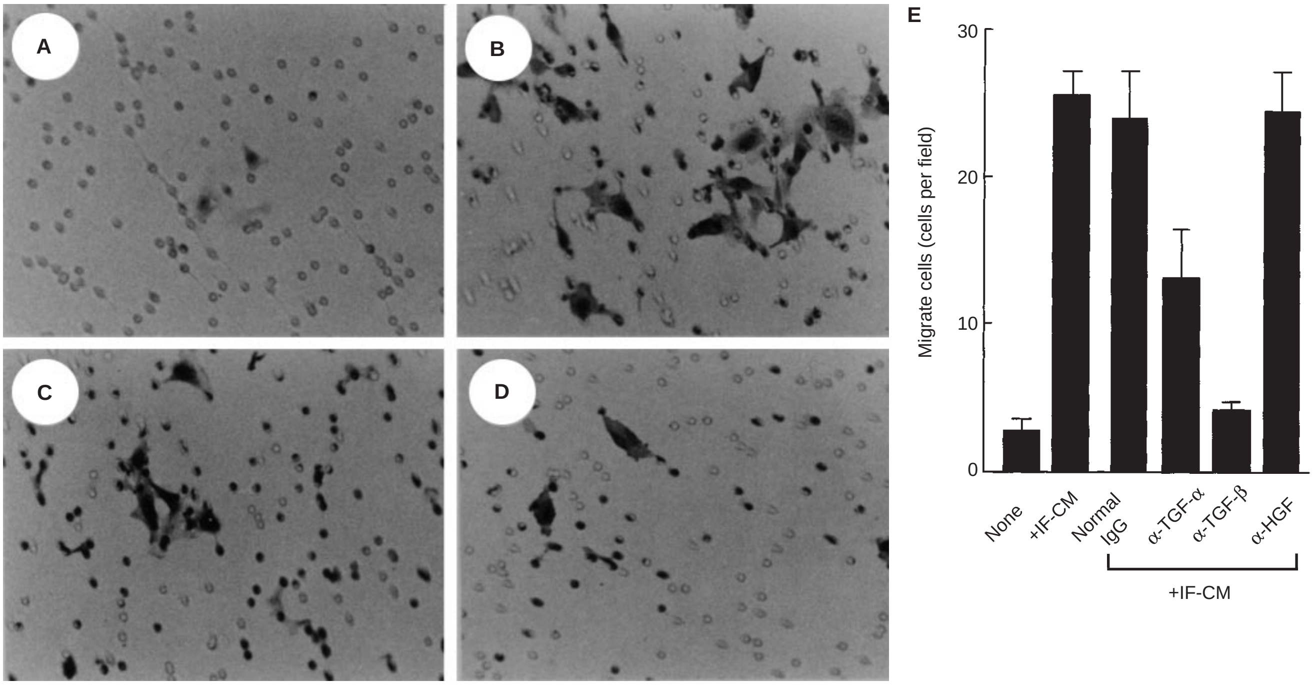

**Figure 2** Inhibitory effect of antibodies on stimulatory effect of IF-derived conditioned medium on migration of GB-d1 cells. Migration of GB-d1 cells was measured using Transwell chamber. (**A–D**) Appearance of cells which migrated through the membrane with pores. GB-d1 cells were cultured in the absence (**A**) or presence of IF-derived conditioned medium (IF-CM) (**B**), IF-CM + anti-TGF-α IgG (**C**), IF-CM + anti-TGF-β1 IgG (**D**). (**E**) The number of migrated cells. IF-CM was added at 50% (v/v) to cultures of GB-d1 cells and antibodies (IgGs) against TGF-α, TGF-β1 and HGF (α-TGF-α, α-TGF-β1, α-HGF) were added at 10 μg $ml^{-1}$. These antibodies neutralized migration of GB-d1 cells stimulated by 10 ng $ml^{-1}$ TGF-α, TGF-β1 and HGF (not shown)

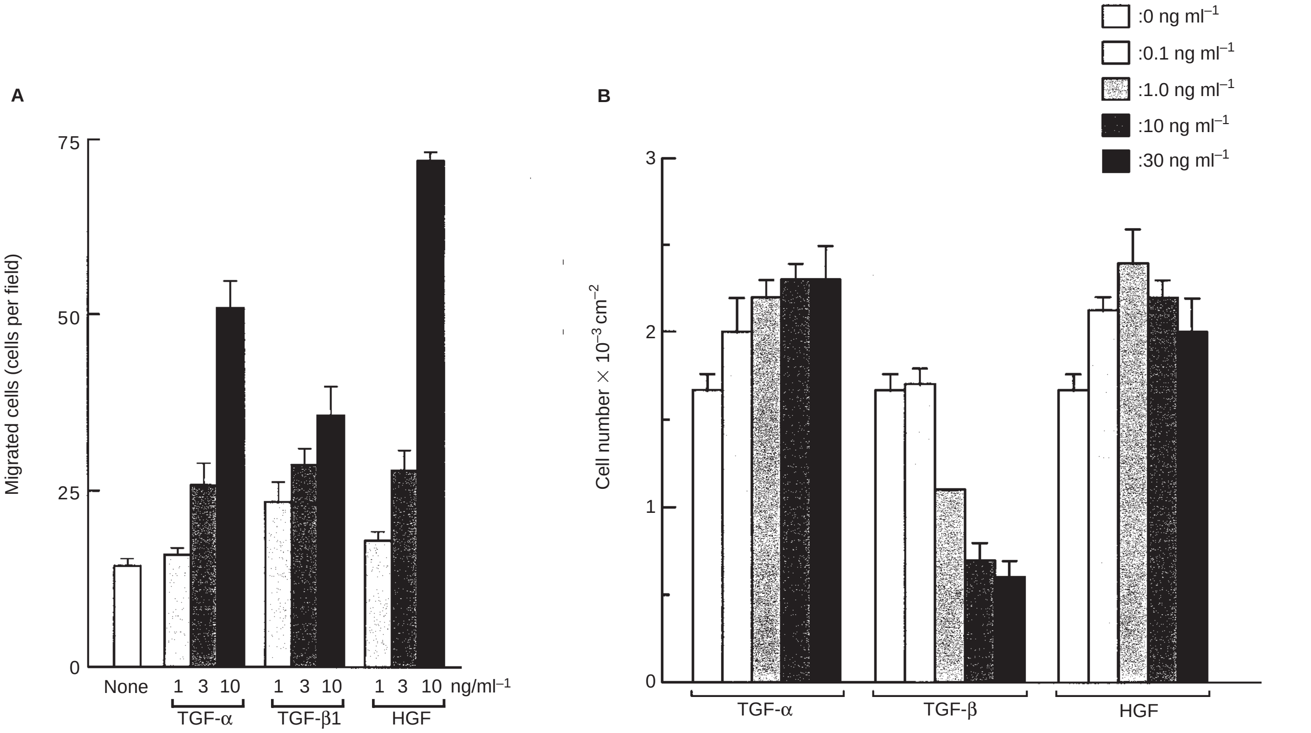

**Figure 3** Effects of TGF-α, TGF-β1 and HGF on migration (**A**) and growth (**B**) of IF cells. Migration of IF cells was measured using Transwell chamber. For cell growth assay, IF cells were cultured in the absence or presence of TGF-α, TGF-β1, or HGF for 72 h athymic mouse, the tumour cells invaded inguinal and axillary lymph nodes, and visible metastatic nodules were present on lungs within 4–6 weeks (manuscript in preparation). To determine if IF human oral squamous cell carcinoma cells produce cell motility factors, conditioned medium derived from IF cells was added to monolayer cultures of MDCK cells and GB-d1 gallbladder carcinoma cells respectively (Figure 1). Addition of conditioned medium from IF cells induced scattering of both MDCK and GB-d1 cells (Figure 1 A–D), indicating that IF cells secrete motility factor(s) bioactive for both MDCK and GB-d1 cells.

*© 1999 Cancer Research Campaign*

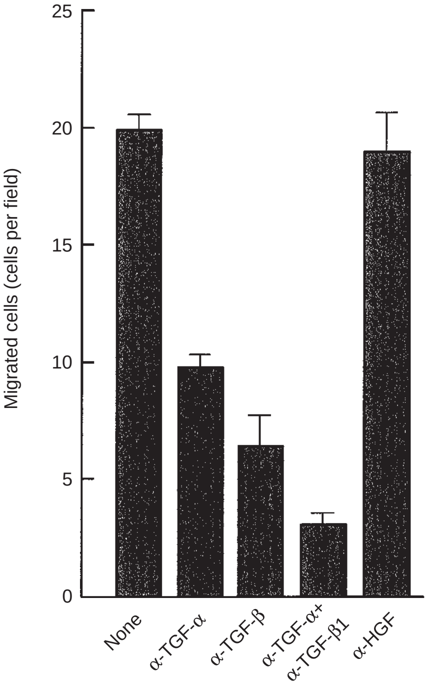

**Figure 4** Effects of antibodies on autonomous migration of IF cells. Migration of IF cells was measured using Transwell chamber and antibodies (IgGs) against TGF-α, TGF-β1 and HGF (α-TGF-α, α-TGF-β1, α-HGF) were added at 10 μg ml⁻¹

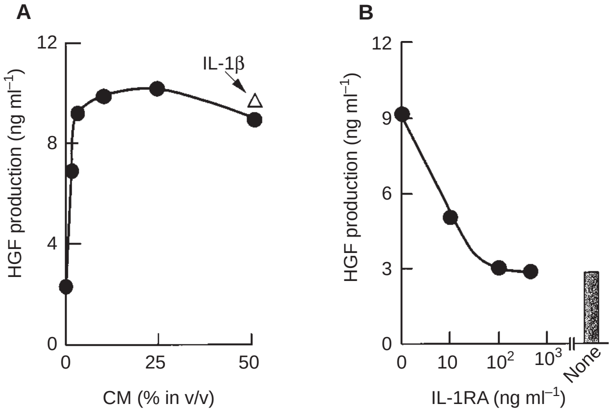

**Figure 5** Enhancement of HGF production in human skin fibroblasts by IF-derived conditioned medium (**A**) and its inhibition by IL-1 receptor antagonist (**B**). Human skin fibroblasts were cultured in the absence or presence of IF-derived conditioned medium or 1 ng ml⁻¹ IL-1β and HGF production during 24-h culture of the fibroblasts was measured (**A**). IF-derived conditioned medium was added at 10% (v/v) to cultures of fibroblasts and various concentrations of IL-1 receptor antagonist (IL-1RA) were added (**B**)

To identify motility factor(s) derived from IF cells, we asked if known growth factors and cytokines would induce scattering of GB-d1 cells (Figure 1). Among various growth factors and cytokines tested, TGF-α, TGF-β1 and HGF showed scattering effects on GB-d1 cells (Figure 1 E–G), while PDGF, bFGF, vascular endothelial cell growth factor, IL-1β, IL-2, IL-6 and tumour necrosis factor-α did not induce cell scattering (not shown). We therefore examined effects of antibodies against TGF-α, TGF-β1 and HGF on cell scattering activity in IF-derived conditioned medium. When an antibody against TGF-α or TGF-β1 was added to the conditioned medium of IF cells, cell scattering activity in the medium was significantly inhibited (Figure 1 H, I), whereas anti-HGF antibody had no inhibitory effect on the cell scattering induced by IF-derived medium (Figure 1, J). This finding meant that IF-derived cell motility factors might be TGF-α and TGF-β1.

For further evidence that IF-derived motility factors are TGF-α and TGF-β1, migration of GB-d1 cells was quantitatively analysed using a Transwell apparatus (Figure 2). GB-d1 gallbladder carcinoma cells were seeded on Transwell filter membrane and the number of cells migrating to the undersurface of the membrane through 8-μm pores was counted. When IF-derived conditioned medium was added, the number of migrated cells greatly increased. However, the addition of anti-TGF-α and anti-TGF-β1 antibodies inhibited migration of GB-d1 cells: the inhibitory effect of anti-TGF-β1 antibody was more potent than that of anti-TGF-α (Figure 2E). These results indicate that IF cells do secrete cell motility factors, which proved to be TGF-α and TGF-β1.

### Autocrine regulation of IF cell motility by TGF-β1 and TGF-α

Based on the finding that IF cells secreted TGF-α and TGF-β1 as cell motility factors, we analysed the regulation of IF cell motility by TGF-α, TGF-β1 and HGF, using Transwell assay. When TGF-α, TGF-β1 and HGF were individually added to culture of IF cells the migration of IF cells was dose-dependently stimulated by these growth factors (Figure 3A). Although a significant number of IF cells migrated through the membrane in control culture, migration of IF cells was further stimulated by these growth factors, in a dose-dependent manner. The potential to stimulate migration of IF cells was HGF > TGF-α > TGF-β1. HGF was most potent in stimulating IF cell motility and fivefold enhancement was seen with 10 ng ml⁻¹ HGF. We also examined effects of these growth factors on proliferation of IF cells (Figure 3B). When the cells were cultured in the absence or presence of growth factors for 72 h, both TGF-α and HGF weakly stimulated the proliferation of IF cells, whereas TGF-β1 inhibited IF cell proliferation; therefore HGF and TGF-α are weak mitogens while TGF-β1 inhibits growth of IF cells.

To observe if the motility of IF cells was regulated by these factors in an autocrine manner, effects of antibodies on autonomous migration of IF cells were tested, using Transwell assay (Figure 4). When antibodies against TGF-α and TGF-β1 were added, migration of IF cells was inhibited to 49% and 33% of the control culture respectively. Simultaneous addition of these antibodies inhibited migration of IF cells to lower levels than seen in cases of anti-TGF-β1 or anti-TGF-α antibody alone. However, anti-HGF antibody had no significant effect on the autonomous migration of IF cells. Consistent with this, HGF was not detected in conditioned medium of IF cells by ELISA (not shown). Therefore, although TGF-α, TGF-β1 and HGF stimulate migration of IF cells, TGF-α and TGF-β, but not HGF, act as autocrine cell motility factors which regulate their own cell motility.

### Induction of fibroblast production of HGF by IF-derived factor

Although IF cells do not produce HGF and HGF does not act as an autocrine motility factor in IF cells, we speculated that stromal fibroblasts-derived HGF might affect migration of IF cells in a paracrine manner. We previously showed that several types of

 © 1999 Cancer Research Campaign

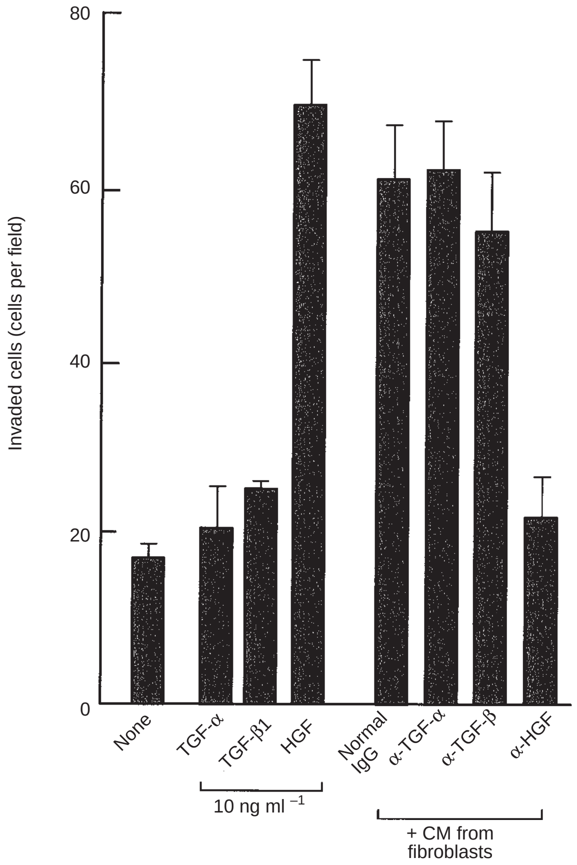

**Figure 6** Effects of TGF-α, TGF-β1, HGF and fibroblast-derived conditioned medium (CM) on invasion of IF cells and inhibition of IF cell invasion induced by fibroblast-derived CM by antibodies. Invasion of IF cells was measured using Matrigel invasion chamber. Growth factors were added at 10 ng $ml^{-1}$, CM from human oral stromal fibroblasts at 50% (v/v) and antibodies (α-TGF-α, α-TGF-β1, α-HGF) were at 10 μg $ml^{-1}$

carcinoma cells secrete inducing factors for HGF production in normal human fibroblasts (Matsumoto et al, 1996; Nakamura et al, 1997). We therefore examined if IF cells would produce an inducing factor for HGF production in normal human fibroblasts derived from dermal tissue. When IF-derived conditioned medium was added to culture of human fibroblasts, HGF production by fibroblasts was dose-dependently stimulated and the maximal stimulatory effect by 4.4-fold was comparable to that seen with 1 ng $ml^{-1}$ IL-1β (Figure 5A). Therefore, IF cells secrete a potent inducing factor for HGF production in fibroblasts.

To identify the IF-derived inducing factor for HGF production, we tested effects of antibodies and antagonistic molecules for inducers of HGF production in fibroblasts, including anti-bFGF, anti-PDGF and an IL-1 receptor antagonist. Addition of an IL-1 receptor antagonist suppressed the inducing activity in IF-derived conditioned medium to close to basal level (Figure 5B), whereas anti-bFGF and anti-PDGF antibodies had no significant effect on HGF-inducing activity in IF-derived conditioned medium (not shown). This finding indicates that IF cells secrete IL-1 (IL-1α, IL-1β1 or both) as an HGF inducer for stromal fibroblast. Similar results were obtained when fibroblasts derived from oral stromal tissue were used, while IL-1β was less potent in stimulating HGF production than that seen with skin fibroblasts (not shown).

### Regulation of IF tumour invasiveness

Our findings indicate that motility of IF cells is regulated by at least three distinct growth factors: TGF-α and TGF-β1 act as autocrine motility factors, while HGF may act as a paracrine motility factor derived from stromal fibroblasts. We then asked whether the invasiveness of IF cells is regulated by these autocrine and paracrine motility factors. IF tumour cells were seeded on Matrigel invasion chamber and cultured for 48 h in the absence or presence of TGF-α, TGF-β1 or HGF (Figure 6). In this apparatus, invasive cells degrade Matrigel extracellular matrix components and then migrate to the undersurface of the membrane. During 48-h culture, a few cells invaded through Matrigel components and a filter membrane in the absence of growth factors. When IF cells were cultured in the presence of HGF, the number of invaded cells greatly increased: fourfold enhancement by HGF. However, both TGF-α and TGF-β1 weakly stimulated invasion of IF cells and their potency in stimulating invasion of IF cells was much less than that of HGF. On the other hand, addition of conditioned medium from human oral stromal fibroblasts also stimulated the invasion of IF cells. The stimulatory effect by this medium was little inhibited by antibodies against TGF-α and TGF-β1 but was greatly suppressed by anti-HGF antibody.

We next analysed invasiveness of IF tumour cells cultured in collagen gel matrix (Figure 7). When IF cells were grown in collagen gel matrix for 6 days in the absence of growth factors, they grew in a cystic structure with no invasive characteristics (Figure 7A, G). In contrast, when the cells were grown in the presence of HGF, they showed invasive and some characteristics of scattering (Figure 7D). Transverse section of these cells showed that IF cells lost cell–cell contact and several cells invaded the collagen gel matrix (Figure 7H). Similarly, the addition of conditioned medium from human oral stromal fibroblasts to cultures of IF cells induced significant invasive characteristics (Figure 7E). In contrast, addition of TGF-α or TGF-β1 did not induce invasive characteristics in IF cells grown in collagen gels (Figure 7B, C). Importantly, the invasive phenotype induced by fibroblast-derived conditioned medium was almost completely prevented by anti-HGF antibody (Figure 7F), but not by anti-TGF-α and anti-TGF-β1 antibodies (not shown). These observations mean that the invasiveness of IF cells is potently stimulated by HGF, but only marginally so by TGF-α and TGF-β1.

### DISCUSSION

Malignant tumours are characterized by unrestrained growth and invasion into surrounding host tissue. Tumour invasion involves the active locomotion of tumour cells into and through host tissue barriers. Various polypeptide growth factors such as epidermal growth factor, PDGF, TGF-α, TGF-β1 and HGF stimulate not only cell proliferation but also chemotactic migration of various types of tumour cells. Likewise, a few classes of proteins which predominantly stimulate the motility of cells have been identified, using assay systems that measure cell motility (Stoker and Gherardi, 1991; Levine et al, 1995; Silletti and Raz, 1996). It is reasonable to consider that these motility factors are responsible for the highly motile behaviour in invasive tumour cells. Using human oral squamous carcinoma cell line, we analysed the regulation of cellular motility and invasiveness by extracellularly acting proteins produced by tumour cells themselves and stromal

*© 1999 Cancer Research Campaign*

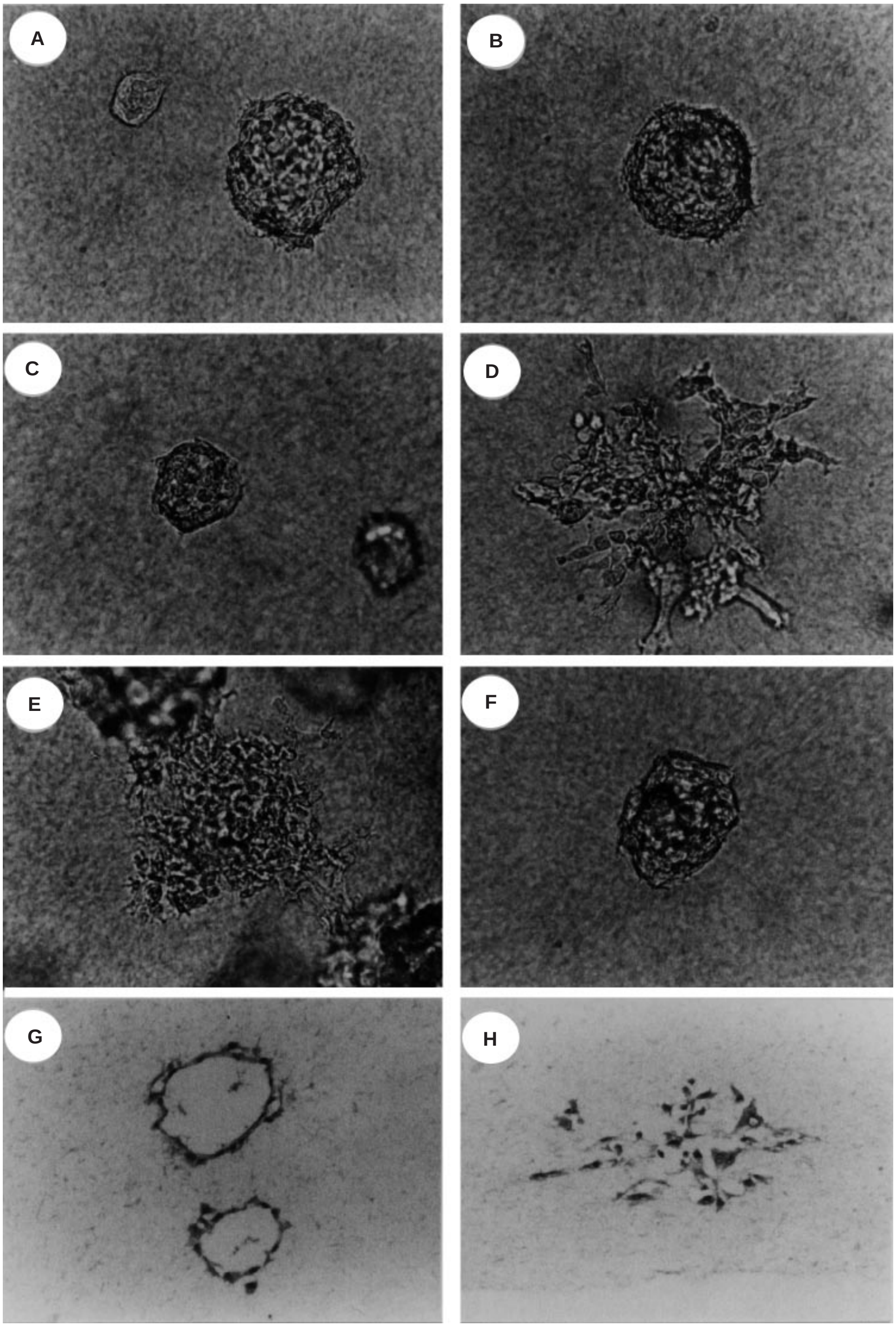

**Figure 7** Invasive growth of IF cells in collagen gel matrix. IF cells were cultured for 7 days in the absence (**A, G**) or presence of TGF-α (**B**), TGF-β1 (**C**), HGF (**D, H**), conditioned medium (CM) from oral stromal fibroblasts (**E**), or fibroblast-derived CM + anti-HGF IgG (**F**). Anti-HGF IgG was added at 10 μg $ml^{-1}$. Appearances of IF cells (**A–F**) and transverse sections (**G, H**) were shown fibroblasts. We identified TGF-α and TGF-β1 to be autocrine-acting motility factors, and HGF to be a stromal-derived paracrine motility factor in IF oral squamous carcinoma cells (Figure 8). Importantly, the invasiveness of IF cells was potently stimulated by HGF, but not by TGF-α and TGF-β1.

TGF-α and TGF-β1 were originally identified as tumour-derived factors which confer anchorage-independent tumour growth, while subsequent studies showed that TGF-α and TGF-β1 are produced in a wide variety of tumour cells and regulate their own growth and motility (Derynck et al, 1987; Sporn et al, 1987; Wright et al, 1993; Wright and Huang, 1996). Several investigators noted that TGF-β1 stimulates migration and invasion of distinct types of tumour cells, through an autocrine or a paracrine manner (Welch et al, 1990; Mooradian et al, 1992; Wright et al, 1993;

*© 1999 Cancer Research Campaign*

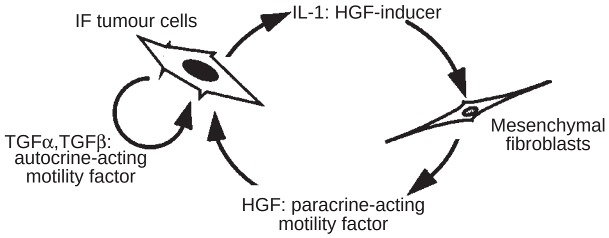

**Figure 8** A schematic representation of the regulation of the IF tumour invasion by autocrine- and paracrine-acting factors. TGF-α and TGF-β1 act as autocrine-acting motility factors, while HGF acts as fibroblast-derived paracrine-acting motility factor. IF cells secrete IL-1 as an inducer for HGF production in fibroblasts

Wright and Huang, 1996; Chicoine and Silbergeld, 1997; Inoue et al, 1997). Likewise, TGF-α stimulates migration and invasion of certain types of tumour cells such as endometrial adenocarcinoma, glioma and uterine cervical carcinoma cells (Ueda et al, 1996, 1997; El Obeid et al, 1997). We identified motility factors derived from IF oral squamous cell carcinoma cells as TGF-α and TGF-β1. Since autonomous migration of IF cells was significantly inhibited by antibodies against TGF-α and TGF-β1, these growth factors may play a role as autocrine-acting cell motility factors which stimulate self migration in IF oral carcinoma cells. On the other hand, HGF stimulates the motility of a diversity of cells, including normal epithelial cells, endothelial cells and a wide variety of tumour cells (reviewed in Jiang et al, 1993, 1998; Zarnegar and Michalopoulas, 1995; Matsumoto et al, 1997). It is noteworthy that, although the motility of IF cells is enhanced by TGF-α, TGF-β1 and HGF, invasion of IF cells is particularly regulated by HGF rather than by TGF-α and TGF-β1. Similarly, we previously noted that the invasiveness of GB-d1 gallbladder cancer cells is potently stimulated by HGF but not TGF-α and TGF-β1, while TGF-α and TGF-β1 both stimulate migration of the cells (Matsumoto et al, 1996). Therefore, cellular responsiveness to stimulatory effects of TGF-α and TGF-β1 on invasion of cancer cells seems to be cell type-dependent. Nevertheless, we speculate that the production of TGF-α and TGF-β1 and autocrine activation of their receptors may be involved in invasive and metastatic behaviour in IF cells. Coexistence of TGF-α and TGF-β1 supports anchorage-independent growth in cancer cells (Derynck et al, 1987; Sporn et al, 1987), which is likely to increase metastatic potential. Enhancement of tumour migration by TGF-α and TGF-β1 may possibly increase invasiveness in IF cells, in collaboration with stromal-derived HGF.

Invasiveness of tumour cells involves disruption of cell–cell adhesion, enhanced cycle of detachment and attachment of cell–substrate interaction, breakdown of extracellular matrix components and enhanced cell motility. HGF induces tyrosine phosphorylation of β-catenin (Watabe et al, 1993; Shibamoto et al, 1994; Hiscox and Jiang, 1999) and focal adhesion kinase (Matsumoto et al, 1994). HGF activates Rho small GTP-binding protein (Takaishi et al, 1994; Ridly et al, 1995), by which intracellular cytoskeletal rearrangement occurs. These events are likely to lead to increased cell motility, dissociation of cell–cell interaction and remodelling of cell–substrate interactions. In addition to these events, HGF induces urokinase-type plasminogen activator (uPA) expression (Pepper et al, 1992; Jeffers et al, 1996; Bennett et al, 1997; Date et al, 1998) and stimulates the production of matrix metalloproteinases (MMPs) (Dunsmore et al, 1996; Bennett et al, 1997; Date et al, 1998). Jeffers et al (1996) demonstrated that induction of the uPA proteolysis network by HGF is coupled to enhanced tumorigenicity and invasive and metastatic properties in certain tumour cell lines. Since the induction and/or activation of proteinases that break down extracellular matrix components seem to be, at the least, critical for induction of invasive potential also in IF tumour cells, we analysed changes in activities of uPA and gelatinases (MMP-2 and MMP-9) in IF cells treated with TGF-α, TGF-β1 or HGF. However, we could not find specific changes in these proteinase activities that correlate to distinct potentials of TGF-α, TGF-β1 and HGF to stimulate invasiveness of IF tumour cells. Identification of extracellular matrix proteinases responsible for the induction of invasiveness by HGF remains to be addressed. Nevertheless, cooperative activation of multiple events driven by cMet/HGF receptor activation seems to be a cause for particular potency of HGF to stimulate the invasion of IF oral carcinoma cells, as well as other tumour cells.

Several lines of studies indicate that the growth and invasive potential of carcinoma cells are influenced through interaction with host stromal cells (van den Hooff, 1988; Matsumoto et al, 1989; Camps et al, 1990; Wernert, 1997). Indeed, the in vitro invasion of various human oral squamous carcinoma cells into collagen gel matrix was specifically induced by co-cultivation with stromal fibroblasts (Matsumoto et al, 1989) and the fibroblast-derived factor responsible for invasion of oral carcinoma cells proved to be HGF (Matsumoto et al, 1994). Thus these earlier findings apparently coincide with our present results: invasion of IF oral squamous carcinoma cells is particularly stimulated by stroma-derived HGF. Moreover, of interest in this study is that IF cells secrete IL-1 as a potent inducer for production of HGF in stromal fibroblasts. We reported that several types of carcinoma cells secrete inducers for HGF production in fibroblasts and fibroblast-derived HGF stimulates invasion of carcinoma cells. IL-1, bFGF and PDGF were identified as tumour-derived HGF-inducers (Matsumoto et al, 1996; Nakamura et al, 1997). There may be mutual paracrine interactions between IF tumour cells and fibroblasts: IL-1 derived from IF tumour cells activates neighbouring fibroblasts to up-regulate HGF production, while fibroblast-derived HGF in turn affects invasion of IF cells (Figure 8). Moreover, HGF has angiogenic activity (Bussolino et al, 1992; Grant et al, 1993) and breast carcinoma cells capable of producing HGF induced more extensive angiogenesis in vivo than that seen in parental cells incapable of producing HGF (Lamszus et al, 1997). Taken together, HGF seems to increase invasive and angiogenic characteristics in IF tumours through a paracrine interaction.

We used a newly established IF human oral squamous carcinoma cell line which possesses highly invasive and metastatic potentials, and identified distinct cells that affect motility and invasiveness of tumour cells in an autocrine or paracrine fashion. The utilization of multiple cell motility/invasion factors that act in distinct pathways in IF oral squamous carcinoma cells may confer highly invasive and metastatic potentials in microenvironments of tumour. Finally, based on the finding that local interaction between IF cells and fibroblasts, as mediated by HGF, is a mechanism leading to even more invasive events, we propose that disruption of this mutual interaction may be one strategy for prevention of tumour invasion and metastasis. In this context, the application of a newly identified four kringle containing antagonist for HGF (Date et al, 1997, 1998) can be tested for potential therapeutic value in suppressing invasion and metastasis of malignant oral squamous carcinoma cells.

*© 1999 Cancer Research Campaign*

### ACKNOWLEDGEMENTS

We are very grateful to M Ohara for helpful comments. This study was supported by a Research Grant for Science and Cancer from the Ministry of Education, Science, Sports and Culture of Japan, a Research Grant for Cancer Research from the Ministry of Welfare of Japan and Research Grants from Haraguchi Memorial Foundation for Cancer Research, the Ryoichi Naito Foundation for Medical Research, Suzuken Memorial Foundation and Nissan Science Foundation.

### REFERENCES

Bennett JH, Furness J, Atkin P and Speight PM (1997) Scatter factor (SF) regulation of matrix metalloproteinase production by oral carcinoma cells. *J Dental Res* **76**: 2140

Birchmeier C and Gherardi E (1998) Developmental role of HGF/SF and its receptor, the Met tyrosine kinase. *Trends Cell Biol* **8**: 404–410

Bussolino F, DiRenzo MF, Ziche M, Bochietto E, Olivero M, Naldini L, Goudino G, Tamagnone L, Coffer A and Comoglio PM (1992) Hepatocyte growth factor is a potent angiogenic factor which stimulates endothelial cell motility and growth. *J Cell Biol* **119**: 629–641

Camps JL, Chang S, Hsu TC, Freeman MR, Hong S, Zhau HE, von Eschenbach AC and Chung LWK (1990) Fibroblast-mediated acceleration of human epithelial tumour growth in vivo. *Proc Natl Acad Sci USA* **87**: 75–79

Chicoine MR and Silbergeld DL (1997) Mitogens as motogens. *J Neurooncol* **35**: 249–257

Date K, Matsumoto K, Shimura H, Tanaka M and Nakamura T (1997) HGF/NK4 is a specific antagonist for pleiotrophic actions of hepatocyte growth factor. *FEBS Lett* **420**: 1–6

Date K, Matsumoto K, Shimura H, Tanaka M and Nakamura T (1998) Inhibition of tumour growth and invasion by a four-kringle antagonist (HGF/NK4) for hepatocyte growth factor. *Oncogene* **17**: 3045–3054

Derynck R, Goeddel DV, Ullrich A, Gutterman JV, Williams RD, Bringman TS and Berger WH (1987) Synthesis of messenger RNAs for transforming growth factor-α and β and the epidermal growth factor receptor by human tumours. *Cancer Res* **47**: 707–712

Dunsmore SE, Rubin JS, Kovacs SO, Chedid M, Parks WC and Welgus HG (1996) Mechanisms of hepatocyte growth factor-stimulation of keratinocyte metalloproteinase production. *J Biol Chem* **271**: 24567–24582

El Obeid A, Bongcan-Rudloff E, Sorby M, Ostman A, Nister M and Westermark B (1997) Cell scattering and invasion induced by autocrine transforming growth factor-α in human glioma cells in vitro. *Cancer Res* **57**: 5598–5604

Furlong RA, Takehara T, Taylor WG, Nakamura T and Rubin JS (1991) Comparison of biological and immunochemical properties indicate that scatter factor and hepatocyte growth factor are indistinguishable. *J Cell Sci* **100**: 173–177

Gherardi E, Gray J, Stoker M, Perryman M and Furlong R (1989) Purification of scatter factor, a fibroblast-derived basic protein that modulates epithelial interaction and movement. *Proc Natl Acad Sci USA* **86**: 5844–5848

Grant DS, Kleinman HK, Goldberg ID, Bhargava MM, Nickoloff BJ, Kinsella JI, Polverini P and Rosen EM (1993) Scatter factor induces blood vessel formation in vivo. *Proc Natl Acad Sci USA* **90**: 1937–1941

Hiscox S and Jiang WG (1999) HGF/SF regulates the phosphorylation of β-catenin and cell–cell adhesion in cancer cells. *Proc Am Assoc Cancer Res*, in press

Inoue T, Chung YS, Yashiro M, Nishimura S, Hasuma T, Otani S and Sowa M (1997) Transforming growth factor-β and hepatocyte growth factor produced by gastric fibroblasts stimulate the invasiveness of scirrhous gastric cancer cells. *Jpn J Cancer Res* **88**: 152–159

Jeffers M, Rong S and Vande Woude GF (1996) Enhanced tumorigenicity and invasion-metastasis by hepatocyte growth factor/scatter factor met signalling in human cells concomitant with induction of the urokinase proteolysis network. *Mol Cell Biol* **16**: 1115–1125

Jiang WG, Hallett MB and Puntis MC (1993) Hepatocyte growth factor/scatter factor, liver regeneration and cancer metastasis. *Br J Surg* **80**: 1368–1373

Jiang WG, Hiscox S, Matsumoto K and Nakamura T (1999) Hepatocyte growth factor/scatter factor, its molecular, cellular and clinical implications in cancer. *Crit Rev Oncol Hematol* **29**: 209–248

Konishi T, Takehara T, Tsuji T, Ohsato K, Matsumoto K and Nakamura T (1991) Scatter factor from human embryonic lung fibroblasts is probably identical to hepatocyte growth factor. *Biochem Biophys Res Commun* **180**: 765–773

Lamszus K, Jin L, Fuchs A, Shi E, Chowdhury S, Yao Y, Polverini PJ, Laterra J, Goldberg ID and Rosen EM (1997) Scatter factor stimulates tumour growth and angiogenesis in human breast cancers in the mammary fat pads of nude mice. *Lab Invest* **76**: 339–353

Levine MD, Liotta LA and Stracke ML (1995) Stimulation and regulation of tumour cell motility in invasion and metastasis. In: *Epithelial–Mesenchymal Interactions in Cancer EXS* **74**: 157–179

Matsumoto K and Nakamura T (1997) Hepatocyte growth factor (HGF) as a tissue organizer for organogenesis and regeneration. *Biochem Biophys Res Commun* **239**: 639–644

Matsumoto K, Horikoshi M, Rikimaru K and Enomoto S (1989) A study of an in vitro model for invasion of oral squamous cell carcinoma. *J Oral Pathol Med* **18**: 498–501

Matsumoto K, Matsumoto K, Nakamura T and Kramer RH (1994) Hepatocyte growth factor/scatter factor induces tyrosine phosphorylation of focal adhesion kinase ($p125^{FAK}$) and promotes migration and invasion by oral squamous cell carcinoma cells. *J Biol Chem* **269**: 31807–31813

Matsumoto K, Date K, Shimura H and Nakamura T (1996) Acquisition of invasive phenotype in gallbladder cancer cells via mutual interaction of stromal fibroblasts and cancer cells as mediated by hepatocyte growth factor. *Jpn J Cancer Res* **87**: 702–710

Miyazawa K, Tsubouchi H, Naka D, Takahashi K, Okigaki M, Gohda E, Daikuhara Y and Kitamura N (1989) Molecular cloning and sequence analysis of cDNA for human hepatocyte growth factor. *Biochem Biophys Res Commun* **163**: 967–973

Mooradian DL, McCarthy JB, Kommanduri KV and Furcht LT (1992) Effect of transforming growth factor-β1 on human pulmonary adenocarcinoma cell adhesion, motility and invasion in vitro. *J Natl Cancer Inst* **84**: 523–527

Nakamura T, Nawa K and Ichihara A (1984) Partial purification and characterization of hepatocyte growth factor from serum of hepatectomized rats. *Biochem Biophys Res Commun* **122**: 1450–1459

Nakamura T, Nishizawa T, Hagiya M, Seki T, Shimonishi M, Sugimura A, Tashiro K and Shimizu S (1989) Molecular cloning and expression of human hepatocyte growth factor. *Nature* **342**: 440–443

Nakamura T, Matsumoto K, Kiritoshi A, Tano Y and Nakamura T (1997) Induction of hepatocyte growth factor in fibroblasts by tumour-derived factors affects invasive growth of tumour cells: in vitro analysis of tumour-stromal interactions. *Cancer Res* **57**: 3305–3313

Naldini L, Weidner KM, Vigna E, Gaudino G, Bardelli A, Ponzetto C, Narsimhan RP, Hartmann G, Zarnegar R and Michalopoulos GK (1991) Hepatocyte growth factor and scatter factor are indistinguishable ligands for the Met receptor. *EMBO* **10**: 2867–2878.

Okada F, Yamaguchi K, Ichihara A and Nakamura T (1989) Purification and structural analysis of a latent transforming growth factor-β from rat platelets. *J Biochem* **106**: 304–310

Pepper MS, Matsumoto K, Nakamura T, Orci L and Montesano R (1992) Hepatocyte growth factor increases urokinase-type plasminogen activator (u-PA) and u-PA receptor expression in Madin-Darby canine kidney epithelial cells. *J Biol Chem* **267**: 20493–20496

Ridly AJ, Comoglio PM and Hall A (1995) Regulation of scatter factor/hepatocyte growth factor responses by Ras, Rac and Rho in MDCK cells. *Mol Cell Biol* **15**: 1110–1122

Russell WE, McGowan JA and Bucher NLR (1984) Partial characterization of hepatocyte growth factor from rat platelets. *J Cell Physiol* **119**: 183–192

Shibamoto S, Hayakawa M, Takeuchi K, Hori T, Oku N, Miyazawa K, Kitamura N, Takeichi M and Ito F (1994) Tyrosine phosphorylation of β-catenin and plakoglobin enhanced by hepatocyte growth factor and epidermal growth factor in human carcinoma cells. *Cell Adhes Commun* **1**: 295–305

Silletti S and Raz A (1996) Regulation of autocrine motility factor receptor expression in tumor cell locomotion and metastasis. *Curr Top Microbiol Immunol* **213**: 137–169

Sporn MB and Roberts AB (1985) Autocrine growth factors and cancer. *Nature* **313**: 745–747

Sporn MB, Roberts AB, Wakefield LM and de Crombrugghe B (1987) Some recent advances in the chemistry and biology of transforming growth factor-β. *J Cell Biol* **105**: 1039–1045

Stoker M and Gherardi E (1991) Regulation of cell movement: the motogenic cytokines. *Biochem Biophys Acta* **1072**: 81–102

Stoker M and Perryman M (1985) An epithelial scatter factor released by embryo fibroblasts. *J Cell Sci* **77**: 209–223

Takaishi K, Sasaki T, Kato M, Yamori W, Kuroda S, Nakamura T, Takeichi M and Takai Y (1994) Involvement of rho p21 small GTP-binding protein and its regulatory protein in the HGF-induced cell motility. *Oncogene* **9**: 273–279

 © 1999 Cancer Research Campaign

Ueda M, Fujii H, Yoshizawa K, Abe F and Ueki M (1996) Effects of sex steroids and growth factors on migration and invasion of endometrial adenocarcinoma SNG-M cell in vitro. *Jpn J Cancer Res* **87**: 524–533

Ueda M, Ueki M, Morimoto A, Fujii H, Yoshizawa K and Yanagisawa T (1997) Stimulatory effects of EGF and TGF-α on invasive activity and 5′-deoxy-5-fluorouridine sensitivity in uterine cervical carcinoma SKG-IIIb cells. *Int J Cancer* **72**: 1027–1033

Ullrich A and Schlessinger J (1990) Signal transduction by receptors with tyrosine kinase activity. *Cell* **61**: 203–212

van den Hooff A (1988) Stromal involvement in malignant growth. *Adv Cancer Res* **50**: 159–196

Watabe M, Matsumoto K, Nakamura T and Takeichi M (1993) Effect of hepatocyte growth factor on cadherin-mediated cell–cell adhesion. *Cell Struc Function* **18**: 117–124

Wehrle-Haller B and Weston JA (1997) Receptor tyrosine kinase-dependent neural crest migration in response to differentially localized growth factors. *Bioessays* **19**: 337–345

Weidner KM, Behrens J, Vanderkerckhove J and Birchmeier W (1990) Scatter factor: molecular characteristics and effect on the invasiveness of epithelial cells. *J Cell Biol* **111**: 2097–2108

Weidner KM, Arakaki N, Hartmann G, Vanderkerckhove J, Weingort S, Rieder H, Fonatsch C, Tsubouchi H, Hishida T, Daikuhara Y and Birchmeier W (1991) Evidence for identity of human scatter factor and human hepatocyte growth factor. *Proc Natl Acad Sci USA* **88**: 7001–7005

Welch DR, Fabba A and Nakajima M (1990) Transforming growth factor-β stimulates mammary adenocarcinoma cell invasion and metastatic potential. *Proc Natl Acad Sci USA* **87**: 7678–7682

Wernert N (1997) The multiple roles of tumour stroma. *Virchows Arch* **430**: 433–443

Wright JA and Huang A (1996) Growth factors in mechanisms of malignancy: roles for TGF-β and FGF. *Histol Histopathol* **11**: 521–536

Wright JA, Turley EA and Greenberg AH (1993) Transforming growth factor-β and fibroblast growth factor as promoters of tumour progression to malignancy. *Crit Rev Oncog* **4**: 473–492

Zarnegar R and Michalopoulas GK (1995) The many faces of hepatocyte growth factor: from haptopoiesis to hematopoiesis. *J Cell Biol* **129**: 1177–1180

*© 1999 Cancer Research Campaign*
